# OUTPACE Tobacco study protocol: a tobacco-free workplace program for dissemination and implementation in opioid use treatment programs in Texas

**DOI:** 10.1186/s13722-026-00696-w

**Published:** 2026-07-17

**Authors:** Asfand B. Moosa, Maggie Britton, Ammar D. Siddiqi, Isabel Martinez Leal, Tzuan A. Chen, Jennifer A. Minnix, Maher A. Karam-Hage, Paul M. Cinciripini, Mhyank S. Sekhar, Lorraine R. Reitzel

**Affiliations:** 1 Department of Behavioral Science, University of Texas MD Anderson Cancer Center, 1155 Pressler Street, Houston, TX 77030 USA; 2 Department of Health Initiatives, National Jewish Health, 1400 Jackson Street, Denver, CO 80206 USA; 3 School of Medicine, University of California San Francisco, 533 Parnassus Ave., San Francisco, CA 94143 USA; 4 HEALTH Research Institute, University of Houston, 4349 Martin Luther King Blvd., Houston, TX 77204 USA; 5 Department of Psychological, Health, and Learning Sciences, University of Houston, 491 Farish Hall, Houston, TX 77204 USA

**Keywords:** Opioids, Tobacco, Cessation, Cancer prevention, Mixed-methods, Implementation science, Intervention, Tobacco-free workplace program

## Abstract

**Background:**

Tobacco use is exceedingly high among individuals with opioid addiction but not commonly addressed in opioid treatment programs (OTPs) due to a combination of pro-smoking social norms in OTPs, providers’ lack of training, and the complex relationship between chronic pain and tobacco use. Comprehensive tobacco-free workplace programs in OTPs can address tobacco use with evidence-based policies and practices, but work is needed for adaptation to this specific setting. Project aims are to successfully implement, evaluate, and disseminate an adapted workplace intervention in OTPs.

**Methods:**

Called [*O*pioid *U*se *T*reatment *P*rograms *A*dvancing *C*essation *E*fforts] OUTPACE Tobacco, the intervention will include tobacco-free workplace policy implementation, clinical workflow changes, employee education, specialized provider training, and resource provision, including connection with treatment through the MD Anderson Tobacco Quitline at 2 Texas OTPs. A mixed method, hybrid effectiveness-implementation design will guide the work; the Exploration, Preparation, Implementation, and Sustainment framework will guide multiple implementation strategies aimed at integrating tobacco dependence care (e.g., screening, brief intervention, and referral for longer-term care) into the clinical workflow.

**Discussion:**

Success will be measured by pragmatic implementation outcomes of acceptability, penetration, and sustainment; clinical outcomes (e.g., patient quit attempts, abstinence) will be assessed with patient/quitline records. Dissemination activities will include the distribution of an implementation guide and technical assistance provision to other OTPs. Innovation of this protocol includes novelty of application in OTPs and the use of a cancer center’s quitline to deliver tobacco treatment. Results from OUTPACE Tobacco will inform tobacco control implementation science while providing practical guidance about integrating best practice recommendations in treating tobacco use disorder in OTPs; ultimately, similar implementations could mitigate the tobacco use disparities experienced by OTP patients.

**Trial registration:**

Not applicable.

## Background

According to the Surgeon General’s 2024 report on addressing tobacco use disparities, tobacco use remains the leading cause of preventable death, disability, and disease in the United States (U.S.), causing nearly half a million deaths annually [1]. This is despite the success of cancer prevention and control initiatives that have drastically reduced the prevalence of cigarette smoking, the most common form of tobacco use, in the general population from 41.9% in 1965 to 11.7% in 2022 [2]. The high morbidity and mortality associated with tobacco use are largely attributable to its strong causal link to several different diseases, including cancer, heart disease, and lung disease [3]. Although smoking rates have declined overall, the burden of tobacco-related diseases is becoming increasingly concentrated in subpopulations that continue to use tobacco products at elevated rates [1, 4].

Among those who are disparately burdened by the consequences of tobacco use are individuals experiencing opioid use disorders (OUDs). For example, the prevalence of cigarette smoking in this group is estimated at 73%-95%: 6 to 8 times higher than the general population [5, 6]. As a result, individuals with OUDs experience a disproportionate burden of tobacco-related health consequences [7, 8] and constitute a priority group for tobacco intervention and relapse prevention efforts. Their use of non-cigarette tobacco products is also elevated. For example, between 18% and 34% of individuals with OUDs report using an e-cigarette product within the past month [9], which is significantly greater than 4.7% in the general population [10]. The elevated use of e-cigarettes/vapes in this group might reflect attempts to switch from conventional cigarette smoking in an attempt at lung harm reduction. However, although the literature is mixed, some studies suggest that harm reduction approaches like this can result in dual use of both products [11], which may not significantly reduce disease risk [12].

Tobacco use can contribute to the onset of OUD, increase its severity, and act as a barrier to successful treatment [13–15]. Pain is a critical factor underpinning the tobacco use-opioid addiction relationship; pain increases tobacco urges, yet tobacco use exacerbates pain, leading to an ever-increasing and cyclical relationship between perceptions of pain intensity and tobacco urges [16, 17]. Indeed, tobacco use is linked to an increased risk of developing physical pain and having greater pain intensity, which results in promoting and/or intensifying the severity of prescription opioid use and misuse among tobacco users [14, 15, 18–20]. Additionally, people who smoke have higher pain intensity following surgery than their non-smoking counterparts. They also experience a less-than-expected decline in daily opioid use post-surgery, putting them at greater risk for misuse [21]. In addition to intensifying pain, nicotine can induce cross-tolerance to morphine, leading to the need for higher opioid doses [22] and increased opioid reward and reinforcement.

Most (> 60%) patients with OUDs have chronic pain conditions [23]. Patients with chronic pain report smoking to relieve pain (a coping strategy involving distraction), despite that it does not actually relieve pain [24]. While patients who quit using tobacco report decreased pain, tobacco users with chronic pain are more likely to relapse to opioids [25]. Pain is commonly referred to as a major barrier to tobacco cessation for these reasons [17, 24]. Because patients are often not aware of the link between their tobacco use/dependence and the occurrence and intensity of pain [24], it is particularly important to target this group for tobacco cessation interventions at the places where they receive care for their OUDs. It is vital to treat tobacco dependence concurrently with OUDs to reduce opioid misuse, improve outcomes of OUD treatment, and reduce tobacco-related health consequences that disproportionately burden this group [26, 27].

Patients with OUD show a significant interest in quitting tobacco use (> 80% have made > 1 quit attempt) [5, 8, 28–30] and can do so successfully with appropriate and tailored support for typically high levels of nicotine dependence. Yet, patients with OUD experience quit success that is 2-3x lower compared to those without OUD [27, 31–33]. Unfortunately, OUD treatment programs (OTPs; i.e., standalone OUD treatment centers or OUD treatment programs within a healthcare organization) struggle to provide evidence-based tobacco cessation care or support for relapse prevention, despite the chronic, relapsing nature of tobacco dependence [8, 25, 30, 34–36]. Some estimates suggest that < 40% of OTPs provide smoking cessation services and fewer than a third provide pharmacotherapy, with sustainment of services being rare in OTPs that do [37–39]. Consequently, there is an urgent need for capacity-building efforts in OTPs to equip their employees with the knowledge and resources to address patients’ tobacco use, support their quit attempts, and prevent relapse. OTPs are well-positioned to provide tobacco cessation treatment, as the average length of patient treatment is long (relative to more generalized substance use treatment centers), many include inpatient facilities, and they are more likely to have experience with and be open to offering pharmacotherapies, including nicotine replacement therapy [29].

Despite the numerous benefits and potential to offer comprehensive tobacco cessation and relapse prevention services in this setting, there remain several challenges to their implementation. One of the most consequential barriers is provider beliefs that tobacco cessation may jeopardize sobriety from non-nicotine drugs or that patients are not interested in quitting [36], both of which are not supported by the literature [26, 38, 39]. These beliefs, along with the sparse implementation of tobacco-free workplace policies in OTPs [40], in turn, contribute to social norms enabling tobacco use. In fact, some treatment providers engage in smoking with their patients, which can subvert the delivery of tobacco cessation care and is associated with lower provision of cessation services [41]. Other barriers that have been reported include knowledge and training gaps in treating tobacco dependence, high employee turnover, and limited capacity from understaffing [37, 40, 42–44].

According to the literature, the previous implementation of a comprehensive tobacco-free workplace program, inclusive of tobacco-free workplace policy adoption, employee education on tobacco cessation care, specialized trainings (e.g., motivational interviewing and train-the- trainer), screening and intervention workflow integration, and resource provision, yielded success in OTPs in Texas [40]. Specifically, employee tobacco-related knowledge and education receipt improved significantly and providers’ intervention delivery increased for the 5 A’s (*A*sking patients about their smoking status, *A*dvising them to quit, *A*ssessing their willingness to quit, *A*ssisting them in quitting, and *A*rranging for follow-up), behavioral counseling, and medication use from pre- to post- program implementation, with significant increases in the use of nicotine replacement therapy. Many of these program-related gains were sustained through 6 months post-implementation. However, there were notable areas that could be improved in future work. For example, only 65% of employees attended tobacco treatment training, with only one half and one third of the 6 OTPs sending a provider to a Tobacco Treatment Specialist and Motivational Interviewing training, respectively. Besides nicotine replacement therapy, less than half of providers reported delivering treatments for tobacco dependence, such as behavioral counseling (39%) and non-nicotine medications (14%), which aligns with previous research identifying intervention gaps [8, 40]. Thus, capacity building and adapting tobacco-free workplace programming to OTPs are imperative to maximizing program-related gains.

Greater improvements in tobacco cessation and relapse prevention services are still needed in OTPs in Texas to improve health outcomes for patients with OUDs. This protocol paper provides a comprehensive overview of OUTPACE (*O*pioid *U*se *T*reatment *P*rograms *A*dvancing *C*essation *E*fforts) Tobacco, an adaptation of the Taking Texas Tobacco Free (TTTF) tobacco-free workplace program [40, 45–55]. Specifically, it will provide guidance on how program-specific adaptations will be made to accommodate the unique needs of OTPs, outline procedures to successfully implement and disseminate the program statewide and offer a framework for assessing the intervention’s outcomes. Implementation and dissemination of OUTPACE Tobacco are expected to build capacity within OTPs in Texas to provide routine tobacco use assessment screenings and offer robust cessation services. These improvements are intended to bolster the success of OUD treatment and improve patients’ long-term health outcomes by addressing their disproportionate use of tobacco products [5, 6].

## Methods/design

### Institutional approval

The project protocol was approved by the Quality Improvement Assessment Board at The University of Texas MD Anderson Cancer Center on 07/12/2023; work began shortly thereafter.

### The intervention’s core components

**Fig. 1:**
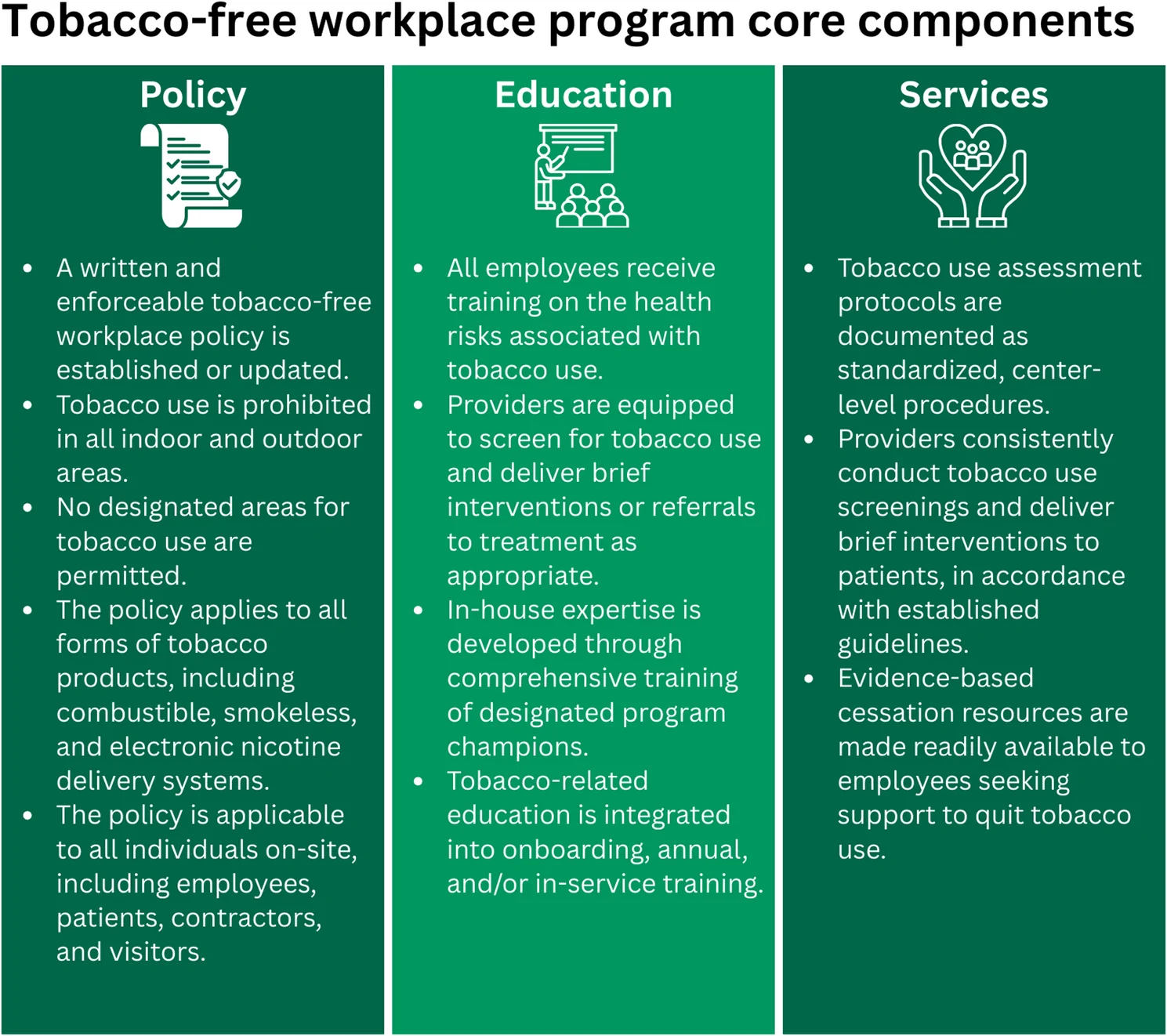
Core components of the tobacco-free workplace program planned for implementation in OUTPACE Tobacco

Our approach leverages a comprehensive tobacco-free workplace program (i.e., TTTF) that has been successfully implemented across a range of healthcare and community-based settings throughout Texas over the past decade, including select OTPs [40]. OUTPACE Tobacco maintains TTTF’s 3 evidence-based core components (see Fig. 1) rooted in: (1) **policy**, (2) **education**, and (3) clinical **services**. However, OUTPACE Tobacco incorporates key insights gained from implementation of TTTF in OTPs [40], allowing for adaptation and optimizing contextual fit in OUTPACE Tobacco, as detailed in the following section, Participatory Approach to Adaptation and Implementation.

### Participatory approach to adaptation and implementation

Adaptation of the tobacco-free workplace program to fit the unique context of OTPs will be essential for successful implementation. To guide this process, the Exploration, Preparation, Implementation, Sustainment (EPIS) framework [56] will be used to identify and respond to contextual differences between OTPs and prior implementation settings (e.g., general substance use treatment settings, mental health facilities), with attention to both inner and outer setting factors and the connections between them.

**Fig. 2:**
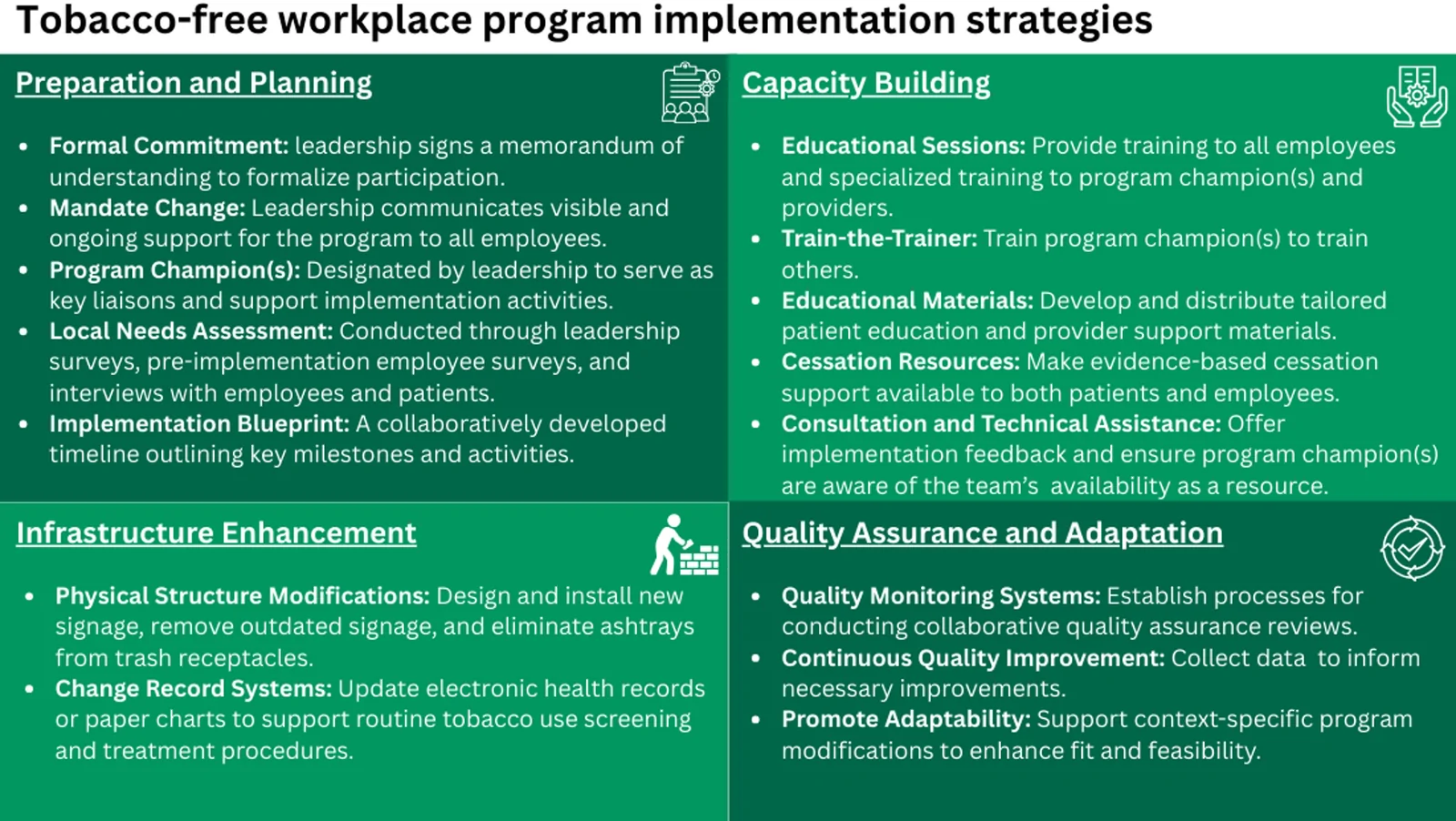
Implementation strategies of the tobacco-free workplace program planned for OUTPACE Tobacco

Planned modifications will be made based on prior implementation of TTTF in this setting and formative input from key stakeholders (i.e., using pre-implementation surveys and qualitative interviews) to proactively tailor implementation strategies (i.e., actions taken to enhance the adoption of the core components; see Fig. 2) to the local context. These modifications will follow the Planned Adaptation approach, which emphasizes balancing adaptations suited to the local context with the maintenance of fidelity to the program’s core components. As implementation progresses, additional reactive changes may be needed, and these will be carefully considered by partners to preserve program fidelity and effectiveness. All modifications—both proactive and reactive—will be systematically documented using the Framework for Reporting Adaptations and Modifications-Enhanced (FRAME) and the Framework for Reporting Adaptations and Modifications to Evidence-based Implementation Strategies (FRAME-IS) [57, 58].

#### Proactive modifications

Proactive modifications are initially informed by prior implementation of TTTF in OTPs, which revealed several implementation gaps including low employee participation in training, insufficient completion of specialized training among providers, and limited delivery of evidence-based pharmacotherapy and behavioral counseling [40]. Additionally, the literature highlights a strong association between pain, opioid use, and tobacco use, underscoring the need for tailored materials that address these intersecting issues [40]. Planned modifications to select implementation strategies (i.e., how the core components are delivered in the OTP; see Fig. 2) will be made accordingly and are outlined in Table 1.

**Table 1:** Planned adaptations to taking Texas tobacco free’s core components and implementation strategies for OUTPACE Tobacco

Implementation Strategy	Taking Texas Tobacco Free Strategy	Strategy Adaptation for OUTPACE Tobacco	Implementation Gaps Addressed
Educational Sessions	90-minute training	1-hour training	Low employee participation in training
	Live online or in-person training	Asynchronous, recorded training option offered	
Cessation Resources	Nicotine replacement therapy starter kit for provider dispensation to patients and employees	Patients and employees access the MD Anderson Quitline for behavioral counseling and nicotine replacement therapy	Insufficient completion of specialized training among providers; limited delivery of evidence-based pharmacotherapy and behavioral counseling
	Specialized training (e.g., on motivational interviewing) to support provider delivery of behavioral counseling	Brief “booster” training on how to refer patients to the MD Anderson Quitline (i.e., deliver the Ask-Advise-Connect intervention)	
Educational Materials	Materials that address tobacco use associations with non-opioid substance use and mental health conditions	Materials that address tobacco use associations with opioid use disorder and pain	Limited relevance of existing materials to unique experiences of individuals with opioid use disorder

Note. The MD Anderson Quitline offers two enrollments per year at no cost to OUTPACE Tobacco partnering OTP patients and employees – each enrollment includes an intake and quit plan session, plus up to six counseling calls and eight weeks of nicotine replacement therapy; Ask-Advise-Connect is an evidence-based brief intervention that outlines the process of Asking about tobacco use, Advising patients to quit, and Connecting them to cessation services via referral (i.e., to a quitline)

The process for making additional proactive adaptations will follow a structured approach informed by the work of Escoffery, Miller, and colleagues, which emphasizes stakeholder input as a central component [57, 59]. Namely, quantitative and qualitative data will be collected from stakeholders (e.g., OTP leadership, employees, and patients) at participating OTPs to inform how best to tailor the program to individual OTP characteristics. These adaptations will account for each OTP’s operational structure, perceived barriers, and identified facilitators. The study team will convene weekly to discuss and evaluate findings and determine potential proactive modifications to be made.

Each OTP will identify one or more “program champions” (i.e., internal employees selected by OTP leadership to serve as key contacts and support implementation efforts). Ideally, program champions will possess the capacity to lead adoption efforts and have demonstrated skills in coordination, planning, and communication, with the role undertaken voluntarily and without financial compensation by the project team. Program champions will collaborate closely with the study team implementor (typically a Health Education Specialist) to ensure adaptations are relevant and feasible for the OTP. Weekly study team meetings will be used to review these ongoing communications and confirm that planned adaptations remain aligned with the core elements of the intervention. Adaptations will be reviewed for consistency with program fidelity, distinguishing between those that preserve core components (i.e., fidelity-consistent changes) and those that represent fidelity-inconsistent changes [57].

#### Reactive modifications

Throughout the implementation phase, the study team implementor will engage with each OTP’s designated program champion through scheduled monthly check-ins to review progress, identify real-time challenges, and discuss any modifications that the OTP may have made independently of prior study team consultation. Additional ad hoc communication may occur as needed to address emerging challenges and provide timely implementation support. These OTP-driven, reactive adaptations will be further reviewed and discussed within the study team using an iterative process to determine alignment with the evidence base and ensure that appropriate documentation is maintained for future evaluation. In cases where reactive changes are considered inconsistent with program fidelity, the implementor will collaborate with the program champion(s) to relay concerns and suggest alternate, evidence-aligned strategies. If the OTP chooses to proceed with an inadvisable approach despite this guidance, the study team will thoroughly document the nature and context of the adaptation for transparency and evaluation purposes.

### Goals and objectives

OUTPACE Tobacco is guided by 3 goals, each supported by a set of specific objectives. The overarching goals are: (1) tailoring our established, evidence-based tobacco-free workplace program in OTPs; (2) carrying out implementation efforts in 2 OTP clinics; and (3) promoting the adapted program more broadly across Texas through the distribution of an implementation guide, relevant resources, and technical assistance to support OTP-led implementation.

#### Goal 1 objectives: adaptation of materials and methods

The objectives associated with Goal 1 are to develop materials to support implementation of OUTPACE Tobacco, including: 1) a tailored training curriculum and focused video/webinar; and 2) *≥* 10 educational materials, including health education materials (e.g., brochures) for patients in English and Spanish and reference tools (e.g., badge cards) for providers in English. Materials are developed based on prior implementation experience, relevant literature, and stakeholder feedback collected during formative evaluation and implementation; 3) the development of a step-by-step implementation guide specific to OUTPACE Tobacco for use in OTPs.

#### Goal 2 objectives: implementation

OUTPACE Tobacco will follow a Hybrid Type 3 mixed methods approach [60], which places primary emphasis on evaluating how the intervention is implemented, while also collecting supplemental data on intervention-related outcomes (such as changes in tobacco use behavior). Since OUTPACE Tobacco comprises core components rooted in evidence-based tobacco control strategies and cessation interventions, the value of this phase lies in understanding how successfully the adapted program is implemented within partnering OTPs.

The implementation objectives were developed based on Proctor’s taxonomy of implementation outcomes [61] and include: (1) the extent to which OTP employees find the OUTPACE Tobacco program implementation acceptable; (2) penetration of education and knowledge to OTP employees; and (3) program sustainment 6-months following the conclusion of formal program implementation, including ongoing education and provision of tobacco-related services. A summary of the implementation objectives and their definitions is provided in Table 2; the section 'Pre- and post-implementation surveys' offers further detail on how these outcomes will be measured.

**Table 2:** Overview of OUTPACE Tobacco’s implementation objectives, desired outcomes, and measurements

Implementation Objective	Operationalization (Desired Outcomes)	Measurements
1. Acceptability	a) Decrease in significant concerns about OUTPACE Tobacco implementation b) *≥* 80% of employees satisfied with implementation	a-b) Pre- and post-implementation employee surveys, and pre-and post-implementation qualitative interviews
2. Penetration	a) *≥* 1 TTS embedded in OTP b) *≥* 1 tobacco treatment training co-delivered by TTS c) *≥* 80% of OTP personnel educated d) *≥* 20% knowledge increase following training	a-c) Study team records d) Pre- and post-training knowledge tests
3. Sustainment*	a) Sustainment scale indicators b) 100% new employees trained with tobacco education curriculum c) 100% of patients are screened for tobacco use d) > 0 patients receive tobacco use intervention	a) Provider REport of Sustainment Scale [100] b-d) Program champion reports c-d) Post-implementation provider surveys

Note. *Desired outcomes measured during the 6-month sustainment period; TTS = tobacco treatment specialist; OTP = opioid treatment program

#### Goal 3 objectives: dissemination

The final set of objectives focuses on the statewide dissemination of OUTPACE Tobacco. Dissemination efforts will focus on OTPs in rural and medically underserved areas of Texas, with a key objective of engaging at least 2, as budget and time allow, additional OTPs in self-guided program implementation. To support these efforts, we will: (1) develop a dedicated webpage to house and promote access to adapted program materials (e.g., patient education resources, provider training videos); (2) actively and passively share OUTPACE Tobacco and its materials/resources with (a) OTPs not involved in Goals 1–2 (i.e., adaptation/implementation), (b) OTP providers, and (c) academic audiences; and 3) offer consultative support and direct technical assistance to OTPs not engaged Goals 1–2.

### Study design

#### OTP recruitment and enrollment

Inclusion criteria for OUTPACE Tobacco’s implementation (Goals 1–2) include: (1) being an OTP in Texas, and (2) having leadership interested in participating and willingness to collaborate on program implementation and relevant data sharing. Potential partner OTPs are identified through several strategies, including engaging previous OTP collaborations (e.g., from TTTF implementation) for warm hand-offs to other OTPs and internet-based searches of licensed OTPs in Texas. Outreach efforts include cold emails, phone calls, participation in professional conferences as a vendor, and distribution of informational one-pagers. Enrollment occurs at the organizational (OTP) level and participation is formalized through a non-binding Memorandum of Understanding by the OTP’s leadership.

#### Participants

Participants involved in Goals 1 and 2 include all employees at the 2 OTPs, encompassing leadership, direct service providers, and non-clinical staff. These participants will be engaged in quantitative data collection activities, with a subset also participating in qualitative data collection procedures. Program champion(s) at each OTP are designated by OTP leadership and serve as the primary liaisons between the OTP and the implementation team, including support for data provision (e.g., organizing stakeholder feedback sessions, provision of monthly clinical services data, etc.). Additionally, OTP patients may be invited to participate in qualitative interviews or focus groups, contingent upon OTP leadership approval. OTP patients often present with multiple socioeconomic concerns and co-occurring mental health conditions [7, 9], though assessment of these factors is outside the scope of this protocol.

**Fig. 3:**
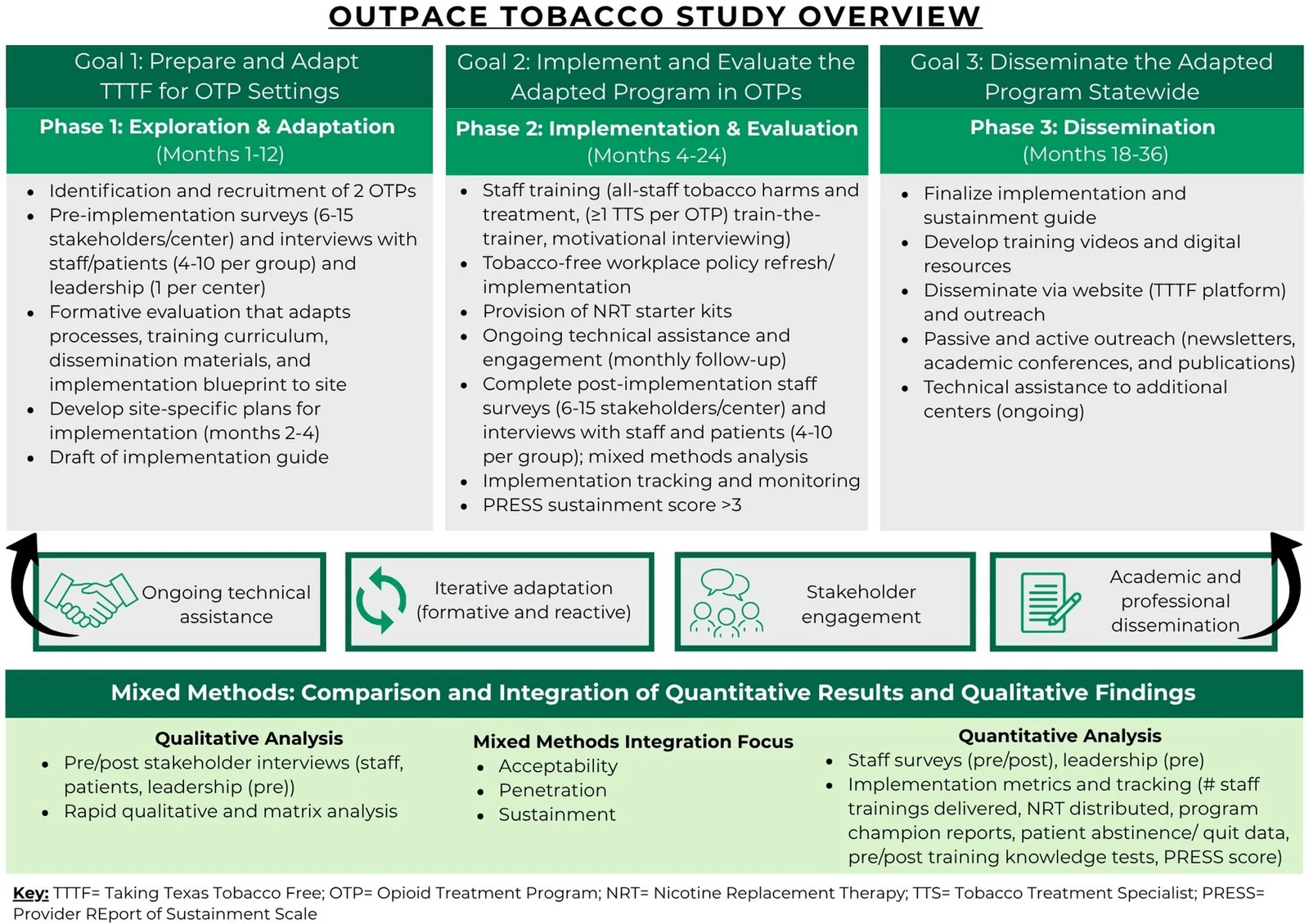
Overview of the OUTPACE Tobacco study design, implementation phases, and evaluation framework

For Goal 3, which focuses on program dissemination, no individual-level data will be collected. Instead, the study team interacts with designated representatives from OTPs not involved in the initial adaptation and implementation phases (i.e., Goals 1–2) to fulfill requests for materials (e.g., educational flyers) and provide technical assistance (e.g., guidance on policy adoption). Data related to Goal 3 activities, such as the number of material orders fulfilled or technical assistance sessions conducted, will be documented and tracked using internal program records (e.g., spreadsheets). A visual overview of the study framework is presented in Fig. 3.

#### Implementation process for participating OTPs

The implementation of OUTPACE Tobacco (Goal 2) is conducted with each participating OTP following enrollment and is guided by the EPIS framework. The active implementation period is expected to last 6–8 months, based on past implementation efforts [40]. However, the timeline may vary depending on OTP’s capacity and needs, as well as anticipating that there may be additional time needed to account for updates to the MD Anderson Quitline infrastructure that were not leveraged in the team’s prior work with OTPs. Concurrently, program materials and methods are adapted using stakeholder feedback and data collected throughout implementation (Goal 1). Table 3 presents key implementation activities (i.e., process components) and their objectives, mapped to corresponding EPIS phases.

**Table 3:** Key implementation activities and associated process objectives of OUTPACE Tobacco

Implementation Process Components ∇		Implementation Process Objectives
E	Conduct pre-implementation quantitative assessments of leadership and employees	Establish a baseline for enrolled OTPs regarding training, knowledge, interventions, implementation concerns, etc., and patient demographics and tobacco use status
E	Conduct pre-implementation qualitative assessments of leadership, employees, and patients (the latter if allowable per OTP)	Understand OTPs’ current practices, barriers, and assets to implementation; guide the adaptation of context-informed interventions within each OTP
E	Create implementation plan for OTP-specific rollout	Negotiate and set an implementation timeline with concrete steps for completion
P (E)	Review Memorandum of Understanding with OTP leadership	Achieve mutual understanding and buy-in for process, timeline, and procedures
P	Select program “champions” (*≥* 1 per OTP) with OTP leadership	Support successful implementation and assessment procedure execution
P	Conduct kickoff meeting with leadership and program champion(s)	Finalize OTP-specific plans and concrete steps for execution
P (S)	Establish/refresh a tobacco-free workplace policy with enforcement and leadership communication plans	Ensure sufficiency of a tobacco-free environment; for centers that rent space, work with them for an acceptable policy/procedure
P (I)	Conduct regular in-person and/or virtual check-ins with champions to discuss the implementation process and advise on challenges that arise	Build and nurture partnership via presence/contact; provide technical assistance; understand implementation processes; identify areas to improve intervention fit
I (S)	Sponsor champions to become Tobacco Treatment Specialists	Embed specialized knowledge into the OTP; equip champions to lead future trainings
I (S)	Change systems (i.e., outline procedures) to facilitate providers’ delivery of Ask-Advise-Connect to refer patients to the MD Anderson Quitline [89–94]	Support regular intervention provision; make free evidence-based care available to patients to decrease barriers to care and enhance cessation; document intervention provision
I (S)	Change systems (i.e., outline procedures) to facilitate employee access to the MD Anderson Quitline	Make free evidence-based care available to employees to decrease barriers to care and enhance cessation
I (S)	Champions (with or observed by study team) provide general tobacco education to all employees	Increase knowledge about the harms of tobacco use, benefits of quitting, and evidence-based care; train-the-trainer for OTP-led trainings
I (S)	Champions (with or observed by study team) provide education to providers about OTP-specific procedures for Ask-Advise-Connect delivery through brief “booster” trainings	Increase knowledge about how to provide tobacco cessation interventions on site; train-the-trainer for OTP-led trainings
I (S)	Provide OTP with monthly feedback reports about screening and referral practices	Bring attention to quality improvement opportunities
I (S)	Design and provide health education materials (e.g., brochures) for patients and reference tools (e.g., badge cards) for providers	Enhance stakeholders’ expectations for assistance and resource knowledge; ease implementation; increase knowledge about pain, tobacco, and opioid interactions
I (S)	Design, supply, and install custom tobacco-free signage	Support the messaging of a tobacco-free workplace; empower community policy enforcement
I	Conduct post-implementation quantitative assessments of leadership and employees	Gather data on implementation to evaluate program acceptability, penetration, sustainment, etc.
I	Conduct post-implementation qualitative assessments of leadership, employees, and patients	Gather data on implementation to evaluate program acceptability, penetration, sustainment, etc.
S	Provide continuing education information regularly (e.g., newsletters, bi-weekly emails)	Equip providers to sustain/reinforce knowledge through free trainings, many offering continuing education credit
S	Prepare OTP with implementation feedback and suggestions to sustain and enhance policy and practice gains	Provide pre- to post-implementation evaluation results; bring attention to quality improvement opportunities; support long-term program sustainment

Note. ∇ includes implementation strategies, evaluation processes, and resources provided, delineated by letter according to the EPIS Framework where E=Exploration, P=Preparation, I=Implementation, S=Sustainment; OTP = opioid treatment program; the MD Anderson Quitline offers two enrollments per year at no cost to OUTPACE tobacco partner OTP patients and employees – each enrollment includes an intake and quit plan session, plus up to six counseling calls and eight weeks of nicotine replacement therapy; Ask-Advise-Connect is an evidence-based brief intervention that outlines the process of Asking about tobacco use, Advising patients to quit, and Connecting them to cessation services via referral (i.e., to a quitline)

#### Data collection from participating OTPs

Data collection for OUTPACE Tobacco is designed to evaluate both the implementation process and key contextual determinants as described in the Social Cognitive Theory (e.g., behavioral, environmental, and interpersonal influences) and the EPIS framework (e.g., inner and outer contextual factors) [44, 62]. A mixed-methods approach, including both quantitative and qualitative data, will be used, particularly to inform Goals 1 and 2, and to a lesser extent, Goal 3. Although the project qualifies as a quality improvement initiative rather than human subjects research, all procedures follow ethical standards, including informed consent and assurances of confidentiality and data security.

##### Tracking logs

The study team maintains detailed records tracking progress across both implementation and dissemination activities. Information documented includes training completion, resource distribution, technical assistance provided, and outreach metrics such as OTPs contacted or engaged.

##### Pre- and post-implementation surveys

OTP *leadership* (expected *n* = 2) will complete a pre-implementation survey administered via Qualtrics, introduced with a cover letter describing the voluntary nature of participation and data usage protocols. Items on this survey, which include primarily investigator-generated questions, assess existing policies and practices related to tobacco cessation, barriers to program adoption, program implementation priorities, patient demographics, and provider capacity. Responses will guide implementation planning (Goal 2) and help tailor program components to the specific setting (Goal 1). Based on our prior work, we anticipate that the participating OTPs will serve between 45 and 350 unique patients annually [40].

The participants at each OTP (*employees*, including healthcare providers and staff), are expected to range in number from 6 to 15 based on our prior work [40], but the final sample size will depend on organizational size and staffing structure. *Employees* will be invited to complete surveys before and after OUTPACE Tobacco’s implementation. Program champions will help facilitate distribution of the survey link within the OTP. Topics will span tobacco-related training exposure, intervention delivery practices, and perceptions of program relevance and impact. In addition to investigator-generated questions, these surveys will incorporate validated measures such as the Acceptability of Intervention Measure (AIM), Intervention Appropriateness Measure (IAM), and Feasibility of Intervention Measure (FIM) [63] prior to implementation, and the Provider REport of Sustainment Scale (PRESS) [64] post-implementation. Employees will receive a $15 e-commerce gift card for each completed survey.

##### Pre-and post-training tests

OTP employees will attend a 60-minute training provided by the study team, co-delivered by the program champion(s). To evaluate training effectiveness, all attendees will complete brief pre- and post-tests that assess knowledge of tobacco use harms, tobacco-free policy benefits, and evidence-based cessation techniques. These tests consist of 10 multiple-choice questions developed by the study team and provide evidence of knowledge gains from the session content.

##### Program champion reports

Reports from program champions will provide ongoing feedback about tobacco cessation clinical service delivery during implementation and through a 6-month sustainment period. These reports include the number of patients screened for tobacco use and those referred to the MD Anderson Quitline, including basic demographics (e.g., sex, gender, race, ethnicity, etc.). Only de-identified, aggregate data will be shared with the OUTPACE Tobacco team, and reports will be compensated at $100 per month, either via an e-commerce gift card or check/direct deposit of funds (following an invoice) to the OTP, depending on OTP leadership preference.

##### MD Anderson Quitline data

The MD Anderson Quitline team will provide summative data to the OUTPACE Tobacco team on referred patients who enroll in services (permitted enrollment is twice per year per patient/employee, similar to the publicly-available Texas Tobacco Quitline), including completion of intake and quit plan sessions, up to six counseling sessions, and receipt of two four-week supplies of nicotine replacement therapy shipped directly to their homes. The MD Anderson Quitline also tracks self-reported and cotinine-verified abstinence at 3- and 7-months post-intake.

##### Group and individual interviews

The purpose of the qualitative component is to explore key stakeholder perspectives on addressing tobacco dependence within OTPs. These insights will help identify potential implementation challenges and facilitators, inform program tailoring, and clarify reasons that may support or hinder adoption of this tobacco-free workplace initiative. We will employ criterion sampling, a purposive strategy that recruits participants based on specific inclusion criteria [65]: a subset of the OTP leadership and employees directly involved in implementation, and - if allowed by leadership - adult patients (18 + years) who speak English and report current or past tobacco use. As a pragmatic quality improvement initiative, the sample size for these procedures may vary based on OTP-level characteristics and participation, but gaining clarity about the stated interview foci provides context for the scope of data collection. In keeping with our prior work, the range of participants is estimated to be 4–10 per qualitative group interview (i.e., staff or patient, pre and post) in each OTP. Based on our study aim, sample specificity, the use of applied established theory, as well as our analysis strategy, the qualitative sample is expected to yield high information power [66]. All interviews will be led by trained qualitative researchers.

Interview guides will be informed by the program’s formative and summative evaluation aims. Pre-implementation interviews will focus on understanding the OTP’s existing policies and practices related to tobacco use, perceived barriers and supports to implementation, and feedback on patient-facing and provider materials. Post-implementation interviews will assess fidelity to program components, OTP-level modifications made, perceived effectiveness, challenges, and suggested improvements for broader dissemination. This process will help ensure the program is both contextually relevant and responsive to real-world applications. Interview durations are expected to range from approximately 20 to 60 min. Group formats are preferred for employees and patients, but individual interviews will be arranged upon request. Program champions at each OTP will assist in identifying eligible participants and coordinating scheduling with support from the study team. A $25 e-commerce gift card will be provided to participants for their time.

#### Dissemination process (Goal 3)

##### Other OTPs

The step-by-step implementation guide (Goal 1) will describe the OUTPACE Tobacco program and its core components to support independent adoption by other OTPs. The resource will include sample policies, workflow suggestions, frequently asked questions, and links to downloadable provider and patient-facing materials hosted on the program website. The guide will be disseminated via email to OTPs throughout Texas and nationally that were not involved in Goals 1–2. These OTPs will be offered optional technical assistance from the study team, including guidance on policy adoption and educational material integration. Dissemination to non-participating OTPs will begin after the guide is finalized and will continue thereafter throughout the remainder of an anticipated 3-year project period.

##### OTP providers

All OUTPACE Tobacco materials, including health education materials (e.g., brochures), provider reference tools (e.g., badge cards), recorded trainings, etc., will be made publicly available on a dedicated webpage. These resources will be promoted through study staff attendance at professional conferences (e.g., as exhibitors), and at provider-focused meetings and workshops. Additional dissemination will occur through team-led communications, including quarterly newsletters and bimonthly continuing education emails. Dissemination will begin once materials are posted and will continue through the 3-year funding period.

##### Academic dissemination

Academic outreach will consist of submitting abstracts and presenting program findings at conferences (e.g., local, state, national), as well as pursuing peer-reviewed manuscript publications. These scholarly dissemination activities will be ongoing throughout the grant and may continue post-funding period to support the broader dissemination of OUTPACE Tobacco implementation outcomes.

### Data analysis

#### Qualitative procedures

Qualitative data analysis will take place after each round of data collection (pre- and post-implementation), allowing findings to directly support iterative implementation and program refinement. We will employ a rapid qualitative analytic approach [67] supported by matrix-based methods [68] to enable timely interpretation of data that can inform both implementation and dissemination planning. This approach aligns well with the needs of Hybrid Type 3 mixed methods designs, where qualitative findings complement and contextualize quantitative measures [69].

Summary templates will be aligned with predefined domains from each interview guide and will include an “additional information” category to capture emergent content not covered by existing domains. To begin, two trained qualitative analysts will independently review a shared set of five transcripts to ensure consistent interpretation and alignment in the summarization approach. The research analysts will come together to refine the templates, adjust domain categories as needed, and ensure consistency in how the data are interpreted. Once consistency is achieved, the remaining transcripts will be summarized independently by analysts into 2–3-page bullet-style documents, integrating illustrative quotes. These summaries will then be entered into OTP- and role-specific matrices to allow systematic comparison across interviews and locations and facilitate the development of key thematic findings [68]. Analyst triangulation will be used throughout to enhance the reliability and credibility of the analysis. The Planning for and Assessing Rigor in Rapid Qualitative Analysis (PARRQA) framework was used to ensure rigor and validity in planning, conducting and reporting rapid qualitative analysis procedures in this study [70].

#### Quantitative procedures

All surveys and knowledge assessments will be anonymous to encourage candid responses. Quantitative data will be primarily analyzed using descriptive statistics (e.g., means, frequencies, standard deviations) and pre-post comparative analyses to assess changes aligned with program objectives. Survey responses will be downloaded from Qualtrics and analyzed in SAS version 9.4 [71]. Due to the anonymous nature of data collection, individual-level matching will not be possible; instead, data will be aggregated and matched and analyzed at the OTP level. Additionally, inferential methods involving comparisons between pre- and post-implementation measures will use independent sample tests, such as t-tests or Mann-Whitney U tests for continuous variables and chi-square or Fisher’s exact tests for categorical variables. Statistical models will control OTP and adjust for potential confounders such as OTP characteristics (e.g., patient volume, staffing levels), as informed by prior research. Effect sizes will be calculated, and statistical significance will be determined at the *p* < 0.05 threshold.

#### Mixed methods integration

A convergent parallel mixed methods design will be used to evaluate the OUTPACE Tobacco program, with qualitative and quantitative data analyzed independently and then merged at the end of each phase. This design supports a comprehensive understanding of program impact, allowing quantitative findings to assess measurable outcomes while qualitative insights illuminate contextual factors, implementation processes, and adaptations [72]. To reduce bias, qualitative analysts will remain blinded to quantitative results until final integration. Data will be merged using techniques such as joint display tables that align findings across methods. During formative evaluation, qualitative data will guide intervention tailoring; during implementation, both data types will be compared to inform program adjustments; and during final evaluation, integrated analysis will help explain observed quantitative trends using qualitative perspectives.

## Discussion

The implementation of OUTPACE Tobacco in OTPs, alongside its planned dissemination, is anticipated to increase the integration of evidence-based tobacco cessation practices into the routine workflows of participating OTPs. The tobacco use rate among individuals in opioid use recovery is remarkably high (73%–95%), identifying them as a priority population for tobacco control and intervention [5, 6]. Despite the detrimental health effects of concurrent tobacco and opioid use [9, 10], as well as evidence showing that tobacco cessation improves opioid treatment outcomes [26, 27], many OTPs still do not intervene to help their patients quit their tobacco use [8, 25, 30, 34]. By embedding comprehensive, evidence-based tobacco-free workplace programs into OUD treatment infrastructure, OUTPACE Tobacco aims to address these gaps in care. This is essential to improve the quality of life for patients who use tobacco and live with chronic pain, as quitting tobacco can improve their symptoms, contribute to a decreased need to use opioids, and positively impact OUD outcomes [73].

A major adaptation that distinguishes OUTPACE Tobacco from prior implementations of the TTTF tobacco-free workplace program is the integration of the MD Anderson Quitline to provide tobacco cessation support for both patients and employees, which addresses the limited time OTP providers had to attend training and provide patients with tobacco use counseling [40]. The MD Anderson Quitline offers Quitline patients (i.e., referrals) who want to quit tobacco with 6 counseling calls, 8 weeks of dual (i.e., patch plus gum or lozenge) nicotine replacement therapy, and a tailored quit tobacco text message program for up to 2 quit attempts/year. While employee training remains a core component of the evidence-based program, OUTPACE Tobacco has been adapted to offer shorter sessions and asynchronous options to better accommodate providers’ demanding schedules. If successful, OUTPACE Tobacco will provide a replicable model for tailoring a tobacco-free workplace program to OTPs and may inform future public health practice and policy to reduce tobacco-related morbidity and mortality among people with OUD.

Potential barriers to the implementation of this protocol include sustaining engagement amid organizational disruptions. Despite a shared recognition of the importance of addressing tobacco use, OTP leadership’s competing priorities can impact program uptake. To address these issues, OUTPACE Tobacco provides a suite of implementation supports, including: (1) using formative evaluation data to refine programming in response to OTP-specific needs; (2) embedding brief, evidence-based interventions like Ask-Advise-Connect into daily practice [74–76]; and (3) offering ongoing technical assistance alongside access to training and educational materials. Additionally, the inclusion of modest incentives will be used in an attempt to maintain interest in evaluation activities. As the project continues, the team remains committed to collaborating closely with each OTP to troubleshoot emerging challenges and deliver practical, flexible solutions that draw from prior tobacco cessation efforts in this setting.

A notable strength of the project is its use of a convergent parallel mixed methods design which enables simultaneous evaluation of implementation processes and program outcomes. Quantitative indicators, such as training participation, offer a glimpse of reach and behavior change, while qualitative insights from interviews with employees, leadership, and patients provide deeper context for understanding the needs of specific OTPs as well as implementation barriers and facilitators. Using multiple trained analysts and systematic analytic frameworks strengthens the credibility and reliability of the qualitative findings. Moreover, the model builds on a foundation of successful prior implementations in similar healthcare environments [40, 45–55], supporting its relevance and scalability. Additionally, because the MD Anderson Quitline is housed within the same department as the majority of the study team, there are unique opportunities for close collaboration with the MD Anderson Quitline team. This alignment facilitates the tailoring of services specifically for patients and employees referred through the program. Namely, this will include specialized training for quitline counselors on addressing tobacco use among individuals with OUD, with attention to the interaction between pain and tobacco use, a tailored text messaging library that will highlight the connections between tobacco use, opioid use, and pain, and - for the first time - a secure referral method will be made available for employees (an option not previously offered to MD Anderson Quitline partners), reflecting the high prevalence of tobacco use among employees in these settings [41]. Finally, proactive text outreach will be implemented for patients and employees who cannot be reached by phone (who may not answer the quitline calls due to not recognizing the incoming phone number). A final key strength of using the MD Anderson Quitline is the availability of robust data on services delivered and abstinence outcomes, which would not be available to the study team if the state quitline were engaged in OUTPACE Tobacco.

Despite these strengths, the project is subject to limitations. The inclusion of non-combustible tobacco products (e.g., e-cigarettes) within a tobacco-free workplace framework presents both potential strengths and challenges. While such products may serve as harm reduction tools for some individuals with opioid use disorder, their inclusion may introduce complexity in program messaging and implementation, particularly in maintaining consistent tobacco-free policies and clearly communicating expectations to employees and patients across participating OTPs [11]. Anonymous surveys cannot track individual respondents over time, which limits the ability to assess individual behavior changes or the specific impact of discrete program components on them. The anonymity of the data collection process further prevents pre-post comparisons at the individual level. Additionally, high employee turnover, a common challenge in OTPs, may affect implementation consistency and program sustainability [42]. Furthermore, the MD Anderson Quitline is not a public resource; launched in 2018, it has been sustained via competitively awarded institutional funds and extramural grants. Therefore, Texas OTPs wishing to replicate implementation of OUTPACE Tobacco will not have access to the MD Anderson Quitline. However, the state-funded quitline, the Texas Tobacco Quitline [77], provides both counseling and dual nicotine replacement therapy (quantities depend on “participant track” enrollment) [78], for up to 2 quit attempts a year, and so can be used in lieu of the MD Anderson Quitline. Although the Texas Tobacco Quitline mirrors some elements of the MD Anderson Quitline, it does not have the same flexibility for adaptations, or data collection benefits that make it ideal for this quality improvement initiative where implementation and intervention outcomes are being assessed. Nevertheless, the wide availability of a comparable and evidence-based treatment resource (i.e., national quitlines in the USA) supports the broad replicability of OUTPACE Tobacco. Finally, as the study will be conducted exclusively in Texas, results may not fully apply to OTPs operating under different conditions or in other regions. Outside of Texas, OTPs can connect with their state-sponsored quitline to determine service offerings [79]. Despite these limitations, OUTPACE Tobacco offers valuable insights into embedding tobacco cessation support within OUD treatment and establishes a strong model for broader application of comprehensive, evidence-based tobacco-free workplace programs in behavioral health settings.

## Conclusions

Enhancing the provision of tobacco cessation care within OTPs is essential to improving long-term health outcomes for individuals in recovery and addressing a major, yet often overlooked, contributor to morbidity and mortality in this population. The OUTPACE Tobacco program offers an evidence-informed framework for adapting, implementing, and disseminating comprehensive tobacco-free workplace programs within behavioral health environments. Findings from this initiative will help address the disproportionately high rates of tobacco use among individuals receiving treatment for OUD and inform future efforts to integrate cessation support into addiction care workflows. Through its phased implementation, mixed methods evaluation, and emphasis on sustainability and scalability, OUTPACE Tobacco lays the foundation for more equitable and holistic care that supports the overall well-being of patients in recovery and strengthens public health outcomes across Texas.

## Data Availability

No datasets were generated or analysed during the current study.

## Abbreviations

OTPs: Opioid Treatment Programs
OUTPACE: Opioid Use Treatment Programs Cessation Efforts
OUDs: Opioid Use Disorders
5As: Asking patients about their smoking status, Advising them to quit, Assessing their willingness to quit, Assisting them in quitting, and Arranging for follow-up
TTTF: Taking Texas Tobacco Free
EPIS: Exploration, Preparation, Implementation, Sustainment
FRAME: Framework for Reporting Adaptations and Modifications-Enhanced
FRAME-IS: Framework for Reporting Adaptations and Modifications to Evidence-based Implementation Strategies
AIM: Acceptability of Intervention Measure
IAM: Intervention Appropriateness Measure
FIM: Feasibility of Intervention Measure
PRESS: Provider REport of Sustainment Scale
